# Recent advances and upgrades in the advanced crystallographic program at NSF’s ChemMatCARS

**DOI:** 10.1063/4.0001169

**Published:** 2025-10-27

**Authors:** Jinxing Jiang, Tieyan Chang, Kevin Lynch, Yu-Sheng Chen

**Affiliations:** 1 University of Illinois at Chicago, Chicago, IL, USA; 2 The University of Chicago, Chicago, IL, USA

## Abstract

NSF’s ChemMatCARS, operated by the University of Chicago and located at the Advanced Photon Source (APS), continues to expand its capabilities in small molecule crystallography. NSF’s ChemMatCARS is undergoing an upgrade to enhance its cutting-edge diffractometers and detectors for advanced crystallographic techniques at 15-ID-B and 15-ID-D hutches. A new 15-ID-B hutch will support Diffraction Anomalous Fine Structure (DAFS) below 5 keV and features a new Mail-In service. 15-ID-D supports high-energy diffraction, in-situ diffraction, quantum crystallography and advanced structural dynamics. As part of this upgrade, we have commissioned Roadrunner, a high-precision diffractometer equipped with an on-axis camera and fast scanning capabilities. We’ve also deployed the first Pilatus 4 detector at APS, enabling fast, high- quality data collection. Additionally, we’ve developed a Field Programmable Gate Array (FPGA)-based Radio Frequency (RF) divider with fine phase delay for advanced timing experiments. These upgrades significantly enhance the program’s capabilities and accessibility for cutting-edge crystallographic research. This poster will provide an overview of the advanced crystallographic program at NSF’s ChemMatCARS, emphasizing the current capabilities and ongoing developments

